# Approaches to Children’s Exposure Assessment: Case Study with Diethylhexylphthalate (DEHP)

**DOI:** 10.3390/ijerph13070670

**Published:** 2016-07-01

**Authors:** Gary Ginsberg, Justine Ginsberg, Brenda Foos

**Affiliations:** 1 Partnership in Pediatric and Environment Health, Granby, CT 06026, USA; justine1973@hotmail.com; 2 United States Environmental Protection Agency, Office of Children’s Health Protection, Washington, DC 20460, USA; foos.brenda@epa.gov

**Keywords:** children, pregnancy, breast milk, house dust, contaminants, exposure assessment, plasticizer, phthalate, DEHP

## Abstract

Children’s exposure assessment is a key input into epidemiology studies, risk assessment and source apportionment. The goals of this article are to describe a methodology for children’s exposure assessment that can be used for these purposes and to apply the methodology to source apportionment for the case study chemical, diethylhexylphthalate (DEHP). A key feature is the comparison of total (aggregate) exposure calculated via a pathways approach to that derived from a biomonitoring approach. The 4-step methodology and its results for DEHP are: (1) Prioritization of life stages and exposure pathways, with pregnancy, breast-fed infants, and toddlers the focus of the case study and pathways selected that are relevant to these groups; (2) Estimation of pathway-specific exposures by life stage wherein diet was found to be the largest contributor for pregnant women, breast milk and mouthing behavior for the nursing infant and diet, house dust, and mouthing for toddlers; (3) Comparison of aggregate exposure by pathways vs biomonitoring-based approaches wherein good concordance was found for toddlers and pregnant women providing confidence in the exposure assessment; (4) Source apportionment in which DEHP presence in foods, children’s products, consumer products and the built environment are discussed with respect to early life mouthing, house dust and dietary exposure. A potential fifth step of the method involves the calculation of exposure doses for risk assessment which is described but outside the scope for the current case study. In summary, the methodology has been used to synthesize the available information to identify key sources of early life exposure to DEHP.

## 1. Introduction

Exposure assessment is a critical aspect of any chemical evaluation. In some cases, evidence of exposure precedes a complete understanding of chemical effects and so can be the impetus for new toxicology studies or the exploration of exposure sources. For example, the biomonitoring evidence of widespread and increasing human exposure to polybrominated diphenyl ethers (PBDEs) in the 1990s and early 2000s spurred new toxicology and exposure research [1]. This need is especially great when the exposure profile of the chemical intersects with children. Children are a high priority because their toxicokinetic handling and toxicodynamic response to chemicals is often different than the remainder of the population [2,3]. Further, children’s physiological and behavioral factors can make them the most highly exposed sector of the population for chemicals that have widespread distribution. This results from their higher metabolic demands leading to increased food, water and air intake per body weight, as well as behaviors that increase contact with contaminants in soil, house dust and products (e.g., crawling, mouthing behavior) [4,5].

This manuscript’s goals are to [1] describe current concepts in children’s exposure assessment, organizing this information into a broadly applicable methodology; and [2] illustrate the method with a case study for the plasticizer diethylhexylphthalate (DEHP). This chemical was chosen because of its high frequency of detection in children’s environments, as well as having an extensive database that includes biomonitoring, indoor air, house dust, food, and consumer product data [6,7,8,9,10,11]. These exposures may be particularly relevant for early life stages because of the known endocrine disrupting and developmental effects of this plasticizer [12,13]. DEHP’s endocrine disrupting effect has an early life window of heightened vulnerability which can alter male in utero development and have long-term implications for reproductive health [14]. Early life exposures are also associated with an increased tendency for respiratory allergy in children [15]. DEHP exposure assessment is critical to understanding the sources and levels of exposure, as well as the options for decreasing exposure in children [16].

## 2. Types of Exposure Assessment for Children

Risk assessments have traditionally captured children’s exposure through the use of higher contact rates with soil, water and food for the period of childhood for which these rates are relevant. For example, the standard adjustment for children’s soil ingestion rate over the first six years of life is approximately 10 times greater than that assumed for adults on a kg body weight basis [17,18]. However, the purpose of children’s exposure assessment is more than deriving adjustment factors for risk assessment. The following list presents various uses of a children’s exposure assessment including and in addition to risk assessment. It is important to consider the use and purpose of the exposure information in the scoping stage as this may affect the approach and methodology:

I. Exposure assessment for risk assessment: objective is to develop a dose in mg/kg/day (or equivalent) for a specific exposure scenario that involves one or more types of contaminated media. The dose can be specific to a given lifestage such as children, or can include various age groups and be cumulated across the entire lifespan as the average daily dose [19]. This calculation can involve three different approaches: (a) lifetime average daily dose, typically what is used for carcinogenic risk; (b) lifetime average daily dose cumulated over different lifestages; for mutagenic carcinogens this incorporates age dependent adjustment factors (ADAFs) to modify cancer potency [20] and age-specific exposure rates such that certain periods in early life can make a greater contribution to lifetime risk than other periods; (c) average daily dose cumulated over the exposure period being analyzed (e.g., 6 years of childhood or chronic 30 year period as adults). This latter method is often the way exposure is considered for non-cancer risk assessment.

II. Exposure assessment for source apportionment: in some cases calculation of risk may be uncertain or premature due to questions regarding the toxicology database, but there is still an interest in identifying the highest exposure pathways to consider prudent mitigation options. This type of exposure assessment focuses less on the overall dose but rather on identifying key pathways of exposure and the source of chemical to each pathway.

III. Exposure assessment for epidemiology studies: epidemiology studies typically don’t need a dose to correlate exposure with outcome. They need to place individuals into categories of exposure based upon some type of measure (e.g., exposure survey, environmental measurement, biomonitoring) that indicates whether the individual has received a low, medium or high level of exposure [21]. While an estimate of dose per body weight per day can be helpful, it is not required in epidemiology to the same degree as in risk assessment.

IV. Exposure assessment to capture status and trends: measurement of exposure over time can reveal important temporal trends that can be related back to changes in regulations, manufacturing practices and the consumer marketplace. The classic example is the temporal correlation between removal of lead from gasoline and the decline in childhood blood lead [22]. Measurement across different sectors of the population can identify how lifestage, gender, occupation, ethnicity or behavioral factors may affect exposure, while comparison of exposure across different countries can show geographical differences. These descriptive statistics can point out key exposure vulnerabilities. Status and trends assessments are commonly based upon biomonitoring data as opposed to calculations of exposure dose.

V. Exposure assessment to prioritize chemicals, products, sources. The previous types of exposure assessments can be applied to the prioritization of chemicals based upon such considerations as frequency of detection in biomonitoring studies, media where detections occur and concentrations found, whether dose estimates approach or exceed risk-based targets, and trends in exposure over time. The following state governments have prioritized chemicals of concern to children’s health if they meet their definition of toxicity and have evidence of exposure that can include presence in house dust, indoor air, consumer products or detection in biomonitoring studies: Maine [23], Washington [24], Vermont [25] and California [26].

A further consideration is whether one is primarily interested in a particular exposure pathway or in aggregate exposure, the sum across all pathways possible for a given receptor. Aggregate exposure assessment is valuable to assess the relative importance of various pathways, with the estimate of total daily dose of potential use in a risk assessment or within epidemiology studies. Ideally the estimate of aggregate dose can be corroborated between a biomonitoring-based and a pathways-based analysis. In addition to aggregate exposures considered in this analysis, cumulative exposure can also be considered to include exposures across multiple chemicals (for example similarly-acting chemicals such as dioxin congeners, phthalates and within certain classes of pesticides) and non-chemical stressors (for example, lack of access to health care).

## 3. Children’s Exposure Assessment Approach

The development of an exposure assessment that can meet the various purposes described above is aided by following a method that ensures that key developmental windows and relevant exposure pathways are considered. The United States Environmental Protection Agency (USEPA) developed a children’s exposure and risk assessment framework in 2006 and has drafted updated human exposure guidelines [27,28]. A simplified 4 step approach that can be used to evaluate the existing exposure information for a well-studied chemical is outlined below and then applied to DEHP as an illustrative case study.

Four Step Approach for Assessing Children’s Exposure to Environmental Chemicals:

1. Prioritize exposure pathways and age groups for quantitative analysis.
2. Estimate pathway-specific and aggregate exposure.
3. Compare estimate of aggregate exposure from pathways analysis to dose estimates from biomonitoring studies to assess whether results from these different approaches are consistent and thus lead to greater confidence in the overall assessment. Estimates of aggregate exposure can also be compared to toxicity values in a risk assessment as mentioned below.
4. Determine which are the quantitatively most significant exposure pathways and explore which sources of chemical are of importance to these pathways.

A 5th phase is added if conducting a risk assessment, that is to aggregate exposures as doses in a manner compatible with calculation of health risk for cancer (multiplied by the cancer slope factor) or non-cancer (divided by the reference dose (RfD)) depending upon the health outcomes of concern. While central tendency estimates of exposure may suffice for phases 1–4, the risk assessment calculations may also consider the full range of exposures possible to understand whether any members of a particular age group may experience an elevated risk. The scenario and toxicity endpoints being analyzed may also dictate that exposures be calculated for short-term peak exposures rather than a long-term average exposure. If long-term exposure is evaluated, the cumulative dose over different early life stages (e.g., at birth from transplacental, breastfeeding, toddler, school-age, adolescent) may need to be estimated.

## 4. Using the Children’s Exposure Assessment Methodology for DEHP

DEHP is a general purpose high molecular weight phthalate plasticizer used to make polyvinylchloride (PVC) plastic flexible where its content is typically on the order of 30% [29]. PVC polymers have a wide range of uses including in building materials such as wiring and cable coatings, flooring and wall paper, and in consumer products ranging from shoes and shower curtains to vinyl table cloths, carpet backing and furniture upholstery [30,31]. DEHP is also used in a variety of non-PVC applications such as an ingredient in sealants, lacquer and paint. Its use in medical tubing, intravenous bags and similar medical devices has been recognized as an important source of exposure to premature infants and children needing hospitalization, with FDA recommending that alternatives be considered [32,33]. On an acute basis this exposure source can be larger than other pathways analyzed [34,35] and it has been associated with health effects in pediatric patients [32]. However, given that this is a specialized exposure setting that is not widespread across the community, it is not explicitly considered in the current case example.

Of the purposes described above, chemical prioritization, risk assessment, status and trends, and source apportionment, we select the latter, evaluation of pathways and sources, for this case study analysis. DEHP has already been prioritized as having the highest toxicity/exposure rank amongst house dust contaminants assessed in a study focusing on data from France [8]. Biomonitoring studies show that of the phthalate metabolites analyzed those stemming from DEHP were among the highest across the US population including children [36]. An analysis across the Center for Disease Control (CDC) biomonitoring database found that DEHP was one of only a few analytes whose levels indicated a potential exceedance of a health benchmark [37]. In 2014 USEPA prioritized DEHP for further assessment by adding it to its list of Toxic Substances Control Act (TSCA) work plan chemicals [38]. Driving these concerns are the endocrine disruptive effects demonstrated for phthalates in general and DEHP in particular in early life stages [16]. A detailed exposure assessment of DEHP in children is warranted given its widespread detection across the population with indications of higher exposure in young children [6,36], This case example provides an identification of key exposure pathways, an evaluation of aggregate exposure and sources of exposure to children. This information can help inform strategies to mitigate children’s exposures to DEHP if risks are found to be high or if replacement chemicals are identified which have a more favorable toxicology profile.

### 4.1. Phase 1. Scoping Developmental Life Stages and Exposure Pathways for Inclusion in a Detailed Assessment

**Figure 1:**
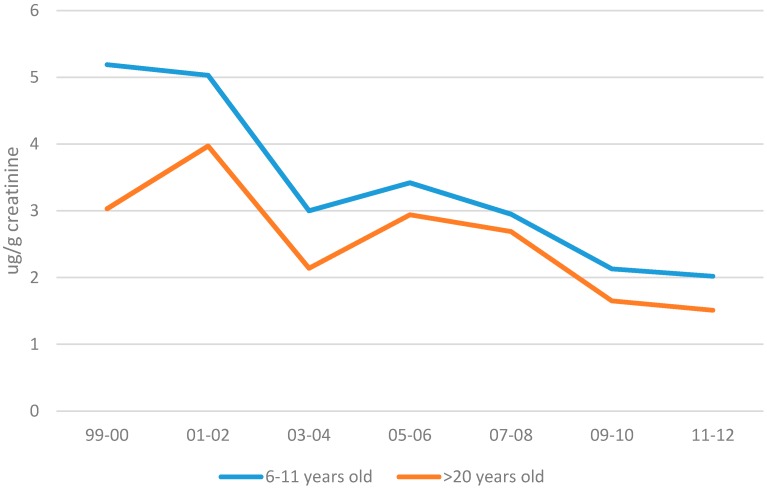
Temporal Trend in MEHP Urinary Concentrations in Children and Adults. Data from CDC Biomontoring Report [50].

The first goal of exposure assessment is to determine the key pathways whereby sensitive early lifestages may contact the subject chemical. This is informed by a series of questions regarding the potential for children to receive DEHP exposure:

- Is DEHP contained in products designed for children or that children frequently use?
  ○ For the most part the answer to this question is no, or at least not as much as in the past. DEHP presence in toys and childcare articles was limited in the US by federal legislation [39] to a nominal level <0.1%. Exempt from this limit is children’s footwear and most forms of clothing. It is also possible that toys and articles produced prior to the setting of limits are still found in homes.
- Is the chemical detected in media children frequently contact?
  ○ Yes, DEHP is present in consumer products that children can contact such as flooring, shower curtains, upholstery and car interiors. House dust studies routinely find it to be the highest phthalate in this medium with levels also detectable in indoor air; however, indoor air levels of DEHP are typically quite low compared to lower molecular weight phthalates.
  ○ DEHP is not tightly bound to plastic and so can be released via physical contact (e.g., a child mouthing a DEHP containing object; wear and tear on flooring and shoes). Another important release mechanism is volatilization. In spite of its low volatility, the high surface area of many PVC items around the home leads to offgassing and accumulation in house dust [40]. Because of DEHP’s low volatility, it will tend to partition into house dust rather than be found as a gas in indoor air. Thus, house dust is the primary storage compartment for DEHP that has been released from the built environment and consumer products [40].
- Is DEHP contained in foods children can be expected to eat? Is it present in breast milk or formula?
  ○ Yes, DEHP was detected in 70% of the 78 breast milk samples reported in a 2011 study from Germany [41] and in over 80% of samples in a 2015 study in Korea [42]. In the German study, DEHP metabolites were found less frequently than parent compound in breast milk (monoethylhexylphthalate (MEHP) 58%, oxidized metabolites 0%). Similarly, a US study of lactating women found less than 10% of milk samples were positive for DEHP oxidized metabolites but parent compound and MEHP were not analyzed in that study [43]. DEHP has been detected with high frequency in infant formula with overall levels comparable to breast milk [41]. DEHP is a common contaminant of the human diet with analysis of food items in Belgium finding that concentrations of DEHP were typically more than an order of magnitude higher than the other phthalates [11]. DEHP’s contamination of foods may stem from its use in food packaging such as metal cans, plastic wrap and storage containers. DEHP can leach from such packaging and enter the food product itself [44]. Further, certain foods may contact DEHP during production or storage prior to consumer purchase.
- Is DEHP a common drinking water contaminant?
  ○ Yes but at low levels. Analysis of drinking water supplies in Portugal found sporadic detection of DEHP with all detections below 1 ug/L [45]. In Mexico City DEHP was among the most commonly detected contaminants with concentrations from groundwater supplies ranging up to 0.23 ug/L and surface waters ranging up to 2.3 ug/L. The higher concentration in surface water is due to DEHP presence in aqueous discharges into rivers which are also used for drinking water [46]. Bottled water was found to contain DEHP up to 1.7 ug/L, with leaching from the plastic bottle possibly contributing to its detection [47]. Data for DEHP in public drinking water in the United States, as summarized by a USEPA six year review of public supply data, found 1.7% of supplies with an exceedance of the DEHP maximum contaminant limit (6 ug/L) and the central tendency of detected concentrations between 1 and 2 ug/L [48]. These detections were in studies that considered artefactual contamination of samples from phthalates present in sampling devices and labware but often these sources can’t be completely ruled out when there are trace detections.
- Has the contaminant been detected in biomonitoring studies? Is there direct biomonitoring evidence in children?
  ○ Yes, DEHP is detected in urine as hydrolyzed and oxidized metabolites. These biomarkers have relatively short half-lives and so the detected level reflects recent intake. DEHP is present in food, tap water and a variety of consumer products suggesting its intake may be fairly consistent from one day to the next which would make the urinary biomarker a reasonable indicator of long-term average intake. However, repeat sampling from the same individuals indicates a relatively weak within subject correlation (interclass correlation coefficient (ICC) of 0.1 to 0.3) [49]. This source of variability will tend to weaken associations in epidemiology studies. However, statistics for DEHP urinary metabolites from large population studies such as the CDC biomonitoring dataset are useful to depict the frequency and range of concentrations in the general population. DEHP metabolites are commonly found in urine at a frequency approaching 100% [43]. The CDC dataset indicates that 6–11 years old children consistently have higher DEHP exposure than adults and that over time the trend has been for decreasing exposure (Figure 1). The CDC dataset does not have information for younger children but as summarized in a later section, limited data for younger children are available elsewhere.

The result of this pathways scoping assessment is that children’s exposure to DEHP can occur via multiple routes including diet, drinking water, consumer products and the indoor environment (e.g., house dust, indoor air). Biomonitoring data suggest that concentrations in older children and adults have been decreasing over the past 12 years, with this trend compatible with the replacement of DEHP with other plasticizers during this time. However, DEHP exposure still appears to be common with the relative importance of the various pathways in need of definition. It is important to note that the DEHP case study is very data rich, and that in some cases, the life stage based exposure assessment will be undertaken with less information; this is an acceptable practice as long as the limitations of available data are clearly defined.

#### 4.1.1. Target Life Stages

Age windows for exposure assessment are selected based upon changing patterns of exposure and the potential for toxicokinetic and toxicodynamic windows of vulnerability that may be the basis for evaluation of a certain life stage in a risk assessment. USEPA’s guidance on selecting age windows is a useful resource for scoping the exposure assessment [51]. The document recommends age groups based on an understanding of differences in behavior and physiology that may impact exposures in children of various ages; a consistent set of early-life age groups, supported by an underlying scientific rationale, increases the consistency and comparability across exposure assessments.

DEHP is an endocrine disruptor having a specific effect on male development in utero, being capable of causing testicular dysgenesis syndrome. As demonstrated in rats this syndrome can include reduced anogenital distance in males at birth along with the potential for hypospadias (penile birth defect) and reduced sperm count at sexual maturity [52]. Evidence that this effect occurs in humans is consistent with the animal evidence [12,16]. Other long-term consequences from early life DEHP exposure may include disruption of female traits as well as neurodevelopment from in utero exposure [53,54], and longlasting effects on immune system [55] and reproductive organ development [56] from postnatal exposure. Metabolic immaturities may also play a role in early life vulnerability to DEHP. Its metabolic disposition involves esterase cleavage to MEHP, an active metabolite, in intestines, liver and other organs. The active metabolite is oxidized to a series of metabolites that are more readily excreted in urine and that are substrates for conjugation with glucuronide to further facilitate excretion in vitro studies show that the cytochrome P-450s primarily responsible for MEHP oxidation in human tissues are CYPs 2C9 and 2C19 [57]. At birth human liver is immature with respect to the content of these CYPs but develops rapidly in the first months of life; for CYP2C9 adult levels of activity can be reached by 6 months of age while maturation appears to be slower for CYP2C19. During this period CYP2C9 and 2C19 activities are highly variable across individuals [58]. Glucuronidation is also immature at birth and the first several months of life [59]. Therefore, the metabolic clearance processes of CYP-mediated oxidation and Phase II conjugation may be immature in early life and lead to a metabolism-based vulnerability to DEHP. However, this needs further exploration.

#### 4.1.2. Life Stages Targeted in the Current Exposure Assessment

In Utero Development: Pregnancy is selected because it contains the most sensitive window of exposure to DEHP. A masculinization programming window has been identified as the first trimester, weeks 9–14 of human gestation, with this potentially the most sensitive period for DEHP’s effect on in utero male development [12,60]. This suggests that peak exposures to DEHP of just a week or two duration during this masculization window may be sufficient to affect development. Thus, one needs to consider whether there can be episodic sources of exposure during pregnancy.

The main exposure pathways during pregnancy for the general public are dietary, house dust and indoor air. Drinking water exposures are also evaluated. DEHP is not known to be commonly used in personal care products.

Breast-Fed Infant: DEHP’s frequent detection in breast milk makes this a priority lifestage of analysis. Breast fed infants ingest mother’s milk as the major source of fluid and nutrition for 0–6 months postnatal so that is the exposure period chosen for this age group [18]. As stated above, postnatal DEHP exposures may represent an important window of vulnerability to DEHP endocrine effects, as well as a period of reduced metabolic clearance.

The case study for this lifestage considers breast milk ingestion and exposure to indoor air (inhalation, dermal) as the most important pathways to quantitate. We are not including formula-fed infants in this analysis but a similar approach can be used as described herein. The evidence that breast milk and formula have similar DEHP concentrations and the removal of DEHP from baby bottles suggest that exposures to formula-fed and breast-fed infants will be comparable.

Toddler: This lifestage involves behaviors (crawling, exploratory behavior, mouthing) that lead to greater contact with house dust and contaminants in consumer products; this is also a potentially vulnerable life stage to DEHP’s endocrine disruptive effects.

The case study for this lifestage considers diet, house dust ingestion, indoor air (inhalation and dermal), mouthing of objects, dermal contact with shoes and clothing and water ingestion to be pathways for more detailed consideration.

Adolescence: for brevity this age group is not a focus of the current case study. This is a potentially important window for endocrine disruptors due to important developmental events surrounding puberty. Phthalates are known to be present in personal care products, which can be used at a particularly high rate during this age window. However, the type of phthalates used in cosmetics, fragrance, lotions, nail polish and hair products typically does not involve DEHP. The exposure profile for pregnancy may be a reasonable approximation for this age group as well, although as shown in Figure 1, biomonitoring results amongst 6–11 years old children are consistently higher than in adults.

The behaviors associated with the life stages modeled may have a degree of overlap as exemplified by the fact that children may continue to breast feed into the toddler years and ingestion of non-food items may begin before that time. The calculations presented below illustrate the key exposures for pathways which drive the exposure for a particular life stage without including minor pathways.

### 4.2. Phase 2. Calculating Exposure Doses via Individual Pathways and Estimation of Aggregate Exposure

The second portion of the children’s exposure methodology is the estimation of exposure doses via well-defined pathways with estimation of aggregate dose across all pathways. This case study focuses on central tendencies, average or median estimates of exposure. This provides a useful survey of the key pathways and sources. However, for risk assessment a probabilistic approach such as Monte Carlo analysis may be more informative to show a more complete distribution of exposure relative to a health benchmark. The exposure estimates shown are either extracted from other studies as for example DEHP dietary intakes (Table 1), or calculated by us as indicated in the text.

#### 4.2.1. Breast-Fed Infant

USEPA’s Exposure Factors Handbook [18] has a recommended rate of breast milk ingestion that ranges from 510 mL/day (150 mg/kg body wt/day) in the first month of life to 770 mL/day (110 mL/kg/day) in the 3–6 month period with further declines from there. One can consider the ingestion rate for 1–3 month old (690 mL/day or 140 mL/kg/day) as an estimate of the average of this period of peak breast milk ingestion. We have combined this ingestion rate with the median concentration of DEHP + active metabolite MEHP detected in breast milk (6.2 ug/L, [41]) to yield an estimate of breastfeeding exposure of 0.87 ug/kg/day (0.14 L/kg/day × 6.2 ug/L) for 1–3 month old infants. An upper bound estimate based upon the 95th percentile of the DEHP and MEHP detections in breast milk is 23.8 µg/L [41] corresponding to 3.3 ug/kg/day. This is similar to the range of DEHP ingestion calculated by Kim et al. [42] based upon sampling of 62 breast milk samples from Korea, 0.9 to 6.5 µg/kg/day.

Exposure to DEHP in indoor air is a potentially important pathway for this lifestage. Indoor air DEHP concentrations have been measured in studies of homes and day care centers as well as been the subject of indoor fate and transport modeling [40,62,63,64]. A central tendency estimate of 0.1 ug/m^3^ and an upper bound of 1 ug/m^3^ can be derived from Xu et al. [27] based upon modeling results for indoor air checked against a range of results from indoor air studies. We combine this estimate with exposure parameters available for the 0–6 month old infant [46] to derive an inhaled dose of 0.033 to 0.33 ug/kg/day. This calculation used a respiration rate of 4 m^3^/day, an absorption rate of 50% via inhalation and a body weight of 6 kg [18]. This DEHP inhalation dose agrees with estimates provided by Xu et al. [27] for inhalation exposure of young children to indoor air DEHP. Dermal exposure to airborne DEHP can also occur by deposition and partitioning of DEHP into skin followed by transdermal uptake. Xu et al. [40] estimated this to be approximately double the daily dose from inhalation of the same DEHP indoor air concentration. Thus, dermal uptake from vapor phase DEHP can be estimated to be 0.07 to 0.7 ug/kg/day.

Other exposures for this age group are less well defined but may contribute to DEHP exposure, including mouthing of objects. This pathway is discussed in detail in the next section for toddlers. Since data presented in USEPA’s Exposure Factors Handbook [18] suggest mouthing of objects in 3–6 month old occurs at a frequency consistent with older children the pathway estimates for toddlers are applied to this lifestage as well. Since this age is prior to the initiation of crawling, house dust ingestion is not considered to be an important pathway but may not be absent.

The estimates of daily exposure over the first 6 months of life for a breastfeeding infant are summarized in Table 2. These results suggest that breastfeeding is not a key driver of exposure as mouthing of objects can be more important at least for the 3–6 month old infant. Overall, breastfeeding infants are expected to have lower DEHP exposure than toddlers.

Formula-fed infants are not included as a separate analysis because of the evidence cited above that formula and breast milk have similar concentrations of DEHP.

#### 4.2.2. Toddler: 1–2 Years of Age

This age group primarily receives dietary exposure to DEHP from solid foods and nutritive liquids rather than from breast milk. Inhalation and dermal uptake from indoor air can be assumed to be similar to the earlier age estimate as both respiratory rate and body mass will be proportionately increased. However, two new exposure routes, mouthing of non-food objects and house dust ingestion are added. The dietary, house dust, mouthing and drinking water exposures are summarized below.

##### Dietary

DEHP is a common contaminant of the human diet with analysis of 8 phthalates in over 550 different food items in Belgium finding the concentrations of DEHP were typically more than an order of magnitude higher than the other phthalates [11]. This study provided aggregate estimates from all foods for children ranging from 3.4 (50th percentile) to 37.5 ug/kg/day (worst case, highest concentration for each food item) with bread (34% of total), fruits (12.5%) and processed meats (7.4%) being the leading source categories [11]. These calculations involve intake rates per body weight for a variety of food items in children, information that can be obtained from USEPA’s Exposure Factors Handbook [18]. A more detailed analysis of the sources of DEHP in samples of commercial bread found that the packaging material (e.g., plastic vs. paper bag) had less to do with contaminant levels than did factors specific to a particular bakery location [64]. The authors surmise that DEHP contamination of kitchen bakeware and starting ingredients (e.g., flour) are likely to be most important [64]. As shown in Table 1, the Belgium dietary estimates for children are similar to results obtained in studies from Canada, Germany and the US. The Rudel et al. [44] study of San Francisco families was a dietary intervention study in which fresh foods with minimal packaging were replaced for conventional foods over a 3 day trial. The decrease in urinary DEHP metabolite was substantial suggesting that a major portion of dietary intake can come from coatings and packaging used for food items.

##### Drinking Water

Children’s ingestion of DEHP in drinking water was estimated based upon a central estimate drinking water concentration of 2 µg/L in public supplies [48] combined with parameters for this age group: 95th percentile water ingestion rate of 0.89 liter per day for a 11.4 kg body weight [18].

##### House Dust

While phthalate release from flooring, wall treatments, shoes and other household items may occur during normal wear and tear, the driving force for their release is vaporization to indoor air [40]. DEHP has a low vapor pressure but it is loosely bound to plastic polymers and given enough surface area (e.g., as in vinyl flooring), there can be a steady release to indoor air. However, the low vapor pressure causes DEHP to adsorb onto particulates and thus partition into house dust. This reservoir can accumulate to relatively high concentrations. A survey of French residential indoor environments found DEHP to be the highest ranked pollutant based upon concentrations found and potential health effects. The mean house dust concentration was 505 µg/g [8] while a similar mean value was found in a survey of German homes [67]. A compilation of recent US studies found a mean of median DEHP concentrations across studies of 383 µg/g with a maximum detected of 6783 µg/g [68]. A phthalates risk assessment set a default house dust ingestion rate for young children at 100 µg/day [9], somewhat less than the assumption for outdoor soil given that children and the objects they encounter will tend to be less dirty indoors than out. This ingestion rate is also consistent with upper bound values suggested by USEPA for indoor dust ingestion [18]. We calculated house dust DEHP dose from this exposure rate combined with the central and upper bound (maximum) for recent US studies to yield 2.6 to 45 µg/kg/day DEHP ingestion from house dust. An estimate for this exposure pathway was provided by CPSC [10] as being 6.6 ug/kg/day for children between 7 months and 4 years of age. In contrast, a study of Danish children at home and in day care environments estimated a dust ingestion dose of only 0.51 µg/kg/day [7].

Another approach to monitoring of indoor dust was an evaluation of the estrogenic potency of the dust collected at Belgium kindergardens [69]. DEHP was detected in every sample and its concentration was correlated with the estrogenic potency of the samples.

##### Mouthing of Objects

The repetitive placement of nonfood items in the mouth can lead to contaminant exposure both due to the dust/dirt on the object and because of the potential for saliva to dissolve chemicals from the product. DEHP and other phthalates are not tightly bound into the polymer or resin in which they reside and so can be subject to extraction in a child’s mouth. Even though DEHP has been phased out of children’s toys and products, it is still present in a variety of consumer items that children may mouth such as packaging, rainwear, fabric and upholstery. In addition, toys that were sold prior to the phase out my still contain DEHP. Based upon a literature review of mouthing behavior and saliva extraction, Heiland et al. [70] estimated young German children to have on average 3.9 µg/kg/day ingestion of DEHP from mouthing household products with this ranging up to a 95th percentile of 10.8 µg/kg/day [70]. This exposure is expected to be greatest in 0.5–2 years old due to their high degree of exploratory behavior at floor level, although the USEPA Exposure Factors Handbook indicates mouthing behavior frequency in 3–6 month old that is similar to older children [18]. A report by the Consumer Product Safety Commission [10] provided estimates for DEHP mouthing exposure from toys ranging from 10 to 75 µg/kg/day; however, these estimates were from pre-2000 studies and so would not reflect the removal of DEHP from children’s products that has since occurred. These estimates for DEHP are compatible with estimates for DINP, a phthalate plasticizer that has replaced DEHP in many items and for which extensive exposure assessment involving the mouthing pathway has been conducted [71]. The anticipation is that DEHP exposure from mouthing of objects has declined and will continue to do so as a result of the phase-out of DEHP from children’s toys and related products.

##### Dermal Exposure from Clothing and Footwear

Phthalates are a major ingredient of PVC-based sandals and other footwear, and can also be present in clothing, particularly rainwear. CPSC [10] provided estimates of total phthalate intake from dermal absorption from footwear and rainwear to be 17 (low estimate) up to 420 (high estimate) ug/kg/day for 1–3 years old children. These estimates were not specific to DEHP but show that high phthalate exposure is possible from these sources and could be a significant contributor to this exposure assessment if they contain DEHP. Analyses of children’s footwear from the State of Washington described below found most samples to be low in DEHP (*N* = 12).

##### Summary for Toddlers

**Figure 2:**
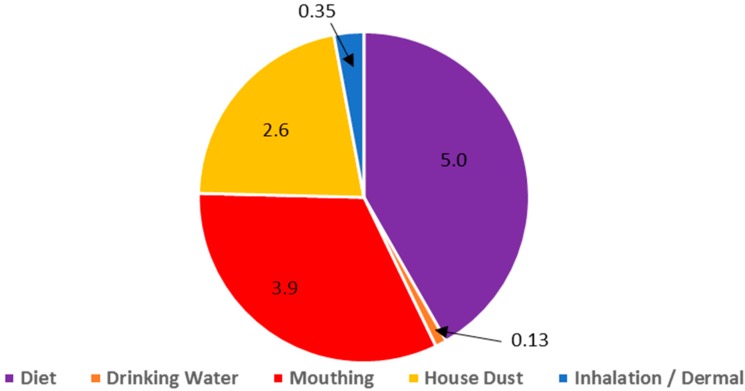
DEHP Exposure Pathways in Toddlers Based Upon Central Estimates of Daily Exposure. Unit: ug/kg/d. Numbers are estimates of dose for each pathway.

Figure 2 and Table 2 present the daily dose of DEHP from different sources, showing that diet and mouthing of objects can have the largest contribution followed by house dust ingestion and then the remaining pathways. The aggregate central estimate exposure for these pathways is 12 µg/kg/day.

#### 4.2.3. Pregnant Women

Adult women will receive DEHP exposure via the diet, indoor air and house dust. A variety of phthalates can be present in personal care products (lotions, deodorant, sunscreen, perfume) but for the most part DEHP is not commonly added as an ingredient to these products. Given that a relatively short window of maximal vulnerability to the in utero endocrine disruption effects of DEHP may exist, it is also worth considering whether there could be large seasonal or day-to-day fluctuations in DEHP exposure.

Dietary exposure is considered the main exposure route in adults [72]. Table 1 shows a variety of estimates of adult dietary intake across several European surveys, with a dietary intervention study in the US providing additional information. The results range from <1 to approximately 5 µg/kg/day with the most robust study, involving 550 food items in Belgium estimating a mean of 1.5 µg/kg/day and an upper bound of 19 µg/kg/day [11]. This set of estimates is used for the current exposure assessment, though there is some uncertainty in this because pregnant women may have different consumption rates and patterns than non-pregnant adults [73].

The inadvertent ingestion of house dust by adults is assumed to occur at a rate of 30 mg/day as per the recommendation in the Exposure Factors Handbook [18]. Based upon a central estimate of 383 µg/g DEHP in house dust (see above) and 30 mg dust ingestion per 73 kg body weight, we estimate 0.16 µg/kg/day house dust DEHP ingestion exposure. As described above for toddlers, the inhaled dose from vaporized DEHP and the dermal absorbed dose are expected to be well below the house dust ingestion dose and thus well below 0.1 µg/kg/day.

Drinking water intake of DEHP is expected to be very low (<0.1 µg/kg/day) based upon a water concentration of 2 µg/L as described above with the 95th percentile ingestion rate of 2.59 liters for a 73 kg body weight for mid-pregnancy [18].

The overall DEHP intake estimate for pregnant women (and adults in general) is thus expected to be 1.7 µg/kg/day, composed primarily of dietary intake. In terms of potential short-term peak exposures that may be relevant to a high vulnerability period of pregnancy, time series data within single individuals indicate an ICC of 0.1 to 0.3 for DEHP suggesting large temporal fluctuations [49]. Since adult exposure is driven by dietary intake, one might consider the upper bound estimate of 19 µg/kg/day identified by Sioen et al. [11] as the maximum exposure possible during a critical window of in utero development for consideration of short-term risk to development. However, this is highly uncertain as more detailed dietary intake data are needed in pregnancy to understand the day to day and week to week variability possible in DEHP intake.

### 4.3. Phase 3. Cross-Check Against Urinary Biomonitoring Estimate of Total Daily Dose

DEHP metabolites have been quantitated across adult populations in Europe and the US with methodologies originally developed by Koch et al. [74] used to convert urinary metabolite data to intake dose. The biomonitoring based quantitation of total exposure provides a useful comparison because it is completely independent of the pathways analysis, relying upon urinary concentrations rather than environmental media concentrations and intake rates. The biomonitoring approach has several uncertainties, such as potential age-related variability in the metabolism and excretion profile of DEHP, whether to use creatinine or urine volume to estimate total DEHP metabolite excretion, and reliability of a single urine sample to be predictive of long-term exposure. However, comparison of aggregate dose estimates via the pathways-based approach and the biomonitoring based approach provides a useful cross-check on the robustness and accuracy of the exposure assessment. If the biomonitoring based approach yields considerably higher dose estimates, then there may be pathways that have been missed or underappreciated. If the pathways-based approach is substantially higher this may reflect different time periods from which the pathways vs. biomonitoring studies were conducted. This is an important consideration for DEHP as exposures have been falling over the past decade (Figure 1); it is also worth considering whether the pathways and biomonitoring based results represent the same percentile of their respective distributions (e.g., comparing medians to medians).

#### 4.3.1. Breastfed Infants

DEHP biomonitoring data for this lifestage were not found and so the pathways-based estimate is the only aggregate dose currently available; thus a Phase 3 comparison between pathways estimate and biomonitoring-based estimate is not possible for this lifestage.

#### 4.3.2. Toddlers

Table 2 provides several biomonitoring-based estimates of total exposure relevant to this age range. The most robust dataset [7] is for somewhat older children but is a useful starting point for this comparison. Beko et al. [7] sampled the urine of 441 3–6 years old Danish children in 2008–2009. This study found that the mean biomonitoring-based dose was 7.4 µg/kg/day (median 4.42, 95th % 16.9). A smaller study of Taiwanese children, aged 2–3 found a similar central tendency 8.1 µg/kg/day [65]. An apparent outlier value is from a small study of children in the US sampled in 2000 which found only 2.8 µg/kg/day (estimate provided in Reference 7). The Beko et al. dataset [7] may provide the best comparison because of its large number of participants and relatively recent sampling period, although the age group is slightly older than what was used for pathways calculations. Comparison of total DEHP exposure by the pathways based (12 µg/kg/day) and biomonitoring-based (7.4 µg/kg/day) approaches yields a close match suggesting a reasonable degree of confidence in the central tendency estimate of DEHP exposure for this age group, especially considering that the slightly younger ages used in the pathways analysis are likely to spend more time mouthing objects as compared to older children [18].

#### 4.3.3. Pregnant Women

Table 2 provides a biomonitoring dataset for adults which converted urinary DEHP metabolites to intake estimates across 209 adults in Belgium [66]. This resulted in an estimate of 1.43 µg/kg/day (50th percentile), ranging up to a maximum of 17.5 µg/kg/day. These estimates closely match the pathways estimates of DEHP exposure in adults (Table 2) suggesting a reasonable degree of confidence in the aggregate and pathways estimates of adult DEHP exposure.

### 4.4. Phase 4. Identification of Key Pathways and Sources of Exposure

DEHP exposure in children and adults has been declining over the past decade but is still considerable and potentially higher than many other common household contaminants. If opportunities are sought for further reductions, a first step is to use the exposure assessment to look upstream at potential sources. The pathway analysis for toddlers, the lifestage with the highest exposure estimate in the current analysis, is summarized in Figure 2. This assessment suggests three areas of primary importance with respect to DEHP exposure in toddlers: diet, mouthing behavior and house dust. These represent three separate source areas for DEHP entry into the environment of a toddler. Similar analyses can be conducted for pregnant women (diet as leading exposure pathway) and breastfeeding infants (mouthing behavior as leading exposure pathway).

#### 4.4.1. Potential Dietary Sources

Food is the predominant source of DEHP exposure in adults and toddlers. DEHP is fat soluble with evidence of its achieving relatively high concentration (several ppm) in high cream content and other fatty foods [75]. Investigation of dairy production found that DEHP most likely enters in several ways including via trace contaminants in grain fed dairy cows, via the machines used for milking, and at the retail level, via the containers used to ship and package milk and other dairy products [76]. In dietary studies, DEHP exposure tended to be greatest from bread, fruit products and processed meats with many foods making small contributions [11]. Similarly, DEHP intake in Norwegian adults was estimated to come from grains and meat products as the two highest source categories [64]. Examination of the sources of DEHP in bread found that it was not related to the packaging used for the bread but was bakery specific, possibly as a result of contamination of flour and/or baking equipment [64]. The dietary intervention study in US families found that packaged foods can be an important source of DEHP in contrast to fresh and unprocessed foods which involved less DEHP and BPA exposure [44].

Overall, these studies do not indicate a particular consumer strategy for reducing DEHP dietary intake although in theory somewhat less exposure may occur by eating lower fat foods and foods involving less processing and packaging. However, DEHP contamination of foods is widespread and at a generally low level so that it would be difficult to single out specific foods or categories of food for reduction.

#### 4.4.2. Potential Contributors to House Dust

DEHP in house dust comes from the many sources of DEHP around the home. Modeling studies have shown that the surface area of vinyl flooring is a key factor in the volatilization release of DEHP into house dust [40]. Other DEHP sources around the home which can contribute high surface area for volatilization are shower curtains, synthetic Christmas trees, vinyl wall coverings, vinyl table cloths and plastic carpet backing. More data are needed regarding the frequency and concentration range for DEHP in these products and whether over time the trend is for the use of replacement phthalates. The ongoing presence of DEHP in house dust could represent old sources from materials that had been purchased years ago and are still in the home. Alternatively, contributions to house dust DEHP may stem from new materials that were recently purchased. Given that young children are especially exposed to chemicals in house dust, the various sources of DEHP merits further investigation. Irregardless of the sources of DEHP in house dust, a simple preventative measure is the frequent cleaning of floors, toys and children’s high contact surfaces to minimize children’s intake of house dust in general. The less house dust on floors and surfaces, the less it will act as a sink for volatilized DEHP [40] and the less that will be available for children’s ingestion. Objects that are mouthed, as discussed in the next section, are also potential contributors of DEHP to house dust.

#### 4.4.3. Potential Sources of Mouthing Exposure

The greatest potential for DEHP mouthing exposure in toddlers is from products designed for children which contain DEHP. However, recent limits on DEHP and several other high concern phthalates from children’s products across the United States and European Union have likely decreased the exposure potential from this pathway. For example a 2014 report prepared by the State of Washington [77] tested a limited number of children’s products for DEHP content. While we do not have results from earlier years, the Washington report stated that the frequency of detection and levels of detection for DEHP in most children’s products were not high, especially in comparison to the DEHP replacement chemical, DINP. For the category “baby accessories” (e.g., teethers, pacifiers, bottles, bibs) only 2 of 38 samples had detectable DEHP, with these concentrations both below 50 µg/g. Out of 18 “bath accessories” DEHP was detected in 5 samples, with one in particular, a bath book, having a relatively high content (1630 µg/g) but other bath books were non-detect and one rubber ducky was found to contain 58 µg/g. Children’s cosmetics had detections in 8 of 26 products with most at relatively low concentration; however, one of three lip gloss samples contained DEHP at 1030 µg/g. Children’s footwear contained DEHP in 5 of 12 samples with only one sample above 100 µg/g (ballet slippers at 336 µg/g). These results suggest that the routine use and mouthing of products intended for children can still lead to DEHP exposure but it will be sporadic, highly variable and typically not at high concentration. In contrast to children’s products, the packaging of items intended for children can have high concentrations of DEHP. The Washington report shows that the plastic packaging and shrink wrap that children’s cosmetics, art supplies, jewelry and toys can contain over 100,000 µg/g DEHP. While such packaging is meant to be discarded after the product is opened, it is possible that the packaging can be a source of DEHP exposure due to mouthing of the item while packaged or because the DEHP leaves the packaging and enters house dust. A 2012 study by an advocacy group in New York State found high levels of DEHP in children’s movie themed backpacks and lunch boxes, as well as in rain boots [78]. Thus, while DEHP sources in children’s products have decreased there may be opportunities to further reduce DEHP contact and mouthing exposures from products and from the packaging of products that are intended for use by children. Other household products may also be a source of children’s mouthing exposure although data on the levels of DEHP in mouthable household items (e.g., clothing, fabric, plastic pens, food packaging containers) are not available.

### 4.5. Risk Assessment Calculations

The exposure estimates provided in this manuscript are to assist with source apportionment, the goal of the current analysis. Such estimates need to be in common units across pathways so that comparisons can be made and the most important exposures identified. These exposures have been described in this analysis as the average dose on a given day of exposure without considering averaging over longer exposure periods, a distribution of exposure percentiles, or different time units as may be needed for risk assessment. Dose calculations in risk assessment are endpoint specific (cancer different than non-cancer). Cancer potency values are based upon a lifetime average daily dose: the animals in the cancer bioassay may have gotten less cancer if they were not dosed for their entire lifespan. The fact that the dosing in animal cancer bioassays doesn’t actually begin until the animals are weaned has led to the development of age dependent adjustment factors (ADAFs) that can account for this extra exposure period and early life vulnerability [20]. Thus, the calculation of exposure dose in children (e.g., breastfeeding infant, toddler) has to be considered within the context of what is the dose over the critical window of heightened cancer risk vulnerability (e.g., the first 2 years of life) so that the estimation of early life cancer risk (exposure dose * adult-based cancer slope factor * ADAF) can be separately calculated and added to the cancer risk from other life stages. For non-cancer endpoints, the assumption is that a chronic period of exposure is necessary to compare the exposure rate to the dose associated with chronic non-cancer risk, the reference dose (RfD). Since less than chronic periods of heightened exposure in early life (e.g., breastfeeding infant, toddler) do not match with the chronic period associated with the RfD, a time weight averaging calculation is often used to relate the exposure scenario back to the RfD. Further details on exposure calculations for risk assessment can be found in documentation provided by USEPA [19].

## 5. Summary and Conclusions

The case study presented for the phthalate plasticizer DEHP provides a 4 step methodology for understanding children’s sources of exposure to a ubiquitous environmental contaminant. DEHP is a good case study because it has sufficient environmental and biomonitoring data to enable comparison of the pathways-based and biomonitoring-based approaches to aggregate exposure assessment in pregnant women and children. A 5th step (calculations for risk assessment) is also described if one is conducting the exposure assessment as part of a DEHP risk assessment. The methodology and its application to DEHP are summarized as follows:

*Prioritize exposure pathways and age groups for quantitative analysis:* the DEHP analysis identified breastfeeding infants, 1–2 years old toddlers, and pregnant women as key life stages for exposure assessment. Pathways specific to each lifestage were identified for quantitative analysis. Other early life periods may also represent important windows of vulnerability or may be important to add into a cumulative exposure and risk assessment. However, for the purposes of the current case study, the selected age groups illustrate the range of exposure considerations and doses of DEHP for early life periods.

*E*s*timate pathway-specific and aggregate exposure:* Table 2 provides exposure estimates for a variety of exposure pathways for the life stages identified in Step 1. The aggregate exposure estimate is greatest for toddlers followed by breastfeeding infants and then pregnant women. The pathways of greatest potential exposure for toddlers are shown in Figure 2 and Table 2 as being diet, mouthing of objects and ingestion of house dust. The other pathways considered, inhalation of vapor phase DEHP, dermal uptake, and drinking water ingestion, were found to make minor contributions. The pathway breakdown for the other lifestages is shown in Table 2 with diet being the dominant pathway for pregnant women and mouthing of objects the dominant pathway for breastfeeding infants.

*Compare the estimate of aggregate exposure from pathways analysis to dose estimates from biomonitoring studies*: the urinary biomarkers for DEHP exposure have been assessed in a variety of studies with several of these studies converting the biomarker result to total aggregate DEHP intake. These biomonitoring-based estimates of total exposure have been compared to the pathways-based estimates in Table 2 for toddlers and pregnant women. In spite of very different methodologies and a variety of uncertainties in the two approaches, their estimate of total aggregate DEHP exposure was very similar. This provides a reasonable degree of confidence in the exposure estimates for these life stages. These aggregate doses are central tendency estimates; additional analysis would be needed to explore whether the pathways and biomonitoring-based approaches are in agreement for upper bound estimates of exposure, although there is no reason to suspect that they would not. Comparison across different quantitative approaches was not possible for breastfeeding infants due to lack of data.

Determine which are the quantitatively most significant exposure pathways and explore which sources of chemical are important to these pathways: the source apportionment focused upon diet, mouthing of objects and house dust as the most important exposure pathways to early life stages. Source apportionment for these pathways is summarized as follows:

I. Dietary sources are widely varied and require further market basket sampling to evaluate trends and food category contributions to total intake. The suggestion from available data is that DEHP exposure is broadly distributed in the diet with a combination of factors (lipid content of food, processing and packaging of the food) having some influence on DEHP content. No clear consumer strategy for reducing DEHP exposure is evident although further analysis of fresh prepared vs. processed/packaged foods may provide a better indication of the importance of these factors [44].

II. Mouthing of objects, both those intended for children’s use as well as general household objects, can be ongoing sources of early life exposure. In particular, product packaging can have high concentrations of DEHP and can thus be a source of mouthing exposure. High DEHP content is also possible in children’s backpacks and lunch containers. These sources may represent opportunities for reformulating consumer items to decrease children’s contact with DEHP.

III. House dust sources are highly varied and appear to include the products and packaging described above as well as general household items which contain DEHP such as vinyl flooring, wall treatments and consumer items such as shower curtains, tablecloths and plastic Christmas trees. Additional data are needed in these source categories to better determine the most quantitatively important sources. Models developed for DEHP release from flooring need to be adapted to other products to understand the relative importance of different source categories. A simple measure to minimize house dust ingestion exposure is public education surrounding the importance of frequent cleaning of floors and high contact surfaces in homes and day care centers.

The result of this methodology for DEHP is the identification of key pathways of exposure along with opportunities for lowering exposure in early life. It has also identified important data gaps and research needs to improve our understanding of the sources of DEHP exposure to pregnant women and children. When needed this approach can be refined using additional life stages [50] and different percentiles of the exposure distribution to explore the variability in exposure and its implications for risk assessment.

This manuscript focused its case study on a well-studied chemical that has a substantial environmental sampling and biomonitoring database. For less well studied chemicals the approach described above can be used to highlight the data that are available and provide exposure estimates and source apportionment to the extent possible. The approach would also be used to identify critical data gaps that prevent a more comprehensive or higher confidence assessment. It may be possible to make a preliminary determination as to whether children’s exposures are likely to be disproportionate due to the chemical’s presence in breast milk, house dust, foods eaten at higher rate by children, or in consumer products designed for children. Such evidence would further emphasize the need for research into children’s exposure pathways and aggregate exposure to that chemical.

## Figures and Tables

**Table 1:** Dietary Exposure Estimates for diethylhexylphthalate (DEHP) from Various Studies.

Study	Population	Foods Considered	Estimation Method	Exposure Estimate (ug/kg/d)
Sioen et al. 2012 [11]	Preschool children in Belgium	Market basket survey of DEHP content of 550 food items	DEHP content × ingestion rate of food	P50: 3.7 P99 (worst case ^a^): 37
Beko et al. 2013 [7]	3–6 years old German children	No dietary analysis	total intake from urinary biomarker—indoor pathways	Average: 6.8
Rudel et al. 2011 [44]	5 San Francisco families, 20 individuals, broad age range	Dietary intervention for 3 days, avoiding packaged foods	Drop in urinary biomarker during intervention converted to intake dose	3.0
Sioen et al. 2012 [11]	Adults in Belgium	Market basket survey of DEHP content of 550 food items	DEHP content × ingestion rate of food	P50: 1.5 P99 (worst case ^a^): 19
CPSC 2010 [10]	6 month to 4 years old children	Canadian market basket survey of 98 foods, Chan and Meek 1994	DEHP content × ingestion rate of food	5.0
CPSC 2010 [10]	Adults	7 day sampling of diet from 50 adult Germans	DEHP content of food × ingestion rate of food	3.95
Sakhi et al. 2014 [61]	Adults	Norwegian market basket survey of 10 food categories	DEHP content of food × ingestion rate of food	Average: 0.4 95th %: 0.8

^a^ DEHP concentration was maximum detect for each food item.

**Table 2:** Summary of DEHP Exposure Estimates (µg/kg/d) Across Pathways in Comparison to Biomonitoring-Based Exposure Estimates ^a^.

Exposure Pathway	Pregnancy	Breastfeeding	Toddler
Diet	1.5	Not applicable	5.0
Breastfeeding	Not applicable	0.9	Not applicable
Inhalation indoor air	<0.1	0.1	0.1
Dermal indoor air	<0.1	0.25	0.25
House dust ingestion	0.16	Not applicable	2.6
Mouthing objects	Not applicable	3.9	3.9
Drinking water	<0.1	Not applicable	0.13
Pathways Total	1.7	5.15	12.0
Biomarker-based Estimates of Total DEHP Exposure	50th % = 1.43 ^e^ Max = 17.5 ^e^	Not available	7.4 ^b^ 8.1 ^c^ 2.8 ^d^

^a^ All data are central tendency estimates (means, medians) rather than upper percentiles. See text for derivation and sources of estimates for pathways approach; ^b^ Estimate is for a slightly older age group, 3–6 years old, Beko et al. 2013 [7]; ^c^ Estimate for 30 children in Taiwan, 2–3 years of age, samples from 2002 to 2003, Lin et al. 2011 [65]; ^d^ Estimate for 19 children in USA, age range 12 to 18 months, sampled in 2000, based upon calculations provided in Beko et al. 2013 [7]. ^e^ Estimate from urinary metabolites in 209 adults of both genders in Belgium, Dewalque et al. 2014 [66].
